# The New Year

**Published:** 1884-01-20

**Authors:** 


### EDITORIALS AND MISCELLANEOUS.

### *THE NEW YEAR.*

We extend to our readers in this, the first number of the year 1884, our hearty greetings and well wishes for their happiness and success.

Brethren, it has been charged that we Doctors "live on the misfortunes ot our fellow-men." We do not so render it, but rather it should be said, we live by *relieving their misfortunes.* Our wish is that our medical friends may relieve the misfortunes of many this year, and that they may be well paid for it. And now, friends, having thus expressed ourself in your behalf, we intend to prove our good feeling and interest in your welfare by giving you a better Journal the present year than ever before. Will you not then remember us also by prompt and cheerful remittances so that we can the better serve you in return? Will you not also write for us, talk for us and aid us in extending our circulation? "So mote it be!" W.

